# Alcohol intoxication and negative mood similarly affect reward learning but not punishment learning in the Iowa gambling task

**DOI:** 10.1017/jdm.2025.10022

**Published:** 2025-11-17

**Authors:** Jonas Dora, Holly Sullivan-Toole, Catherine Zhang, Morgan Opdahl, Kevin M. King

**Affiliations:** 1 University of Washington, USA;; 2 Department of Psychology and Neuroscience, Temple University, USA; 3 Department of Pediatrics, University of Minnesota System, USA

**Keywords:** Iowa gambling task, alcohol, negative mood, computational modeling

## Abstract

This study investigated how alcohol intoxication and negative mood affect decision-making in the Iowa Gambling Task (IGT) in a high-risk sample of adults who regularly drink alcohol. Using a 2×2 between-subjects design (N=160), we experimentally manipulated alcohol intoxication (target BrAC=.06% vs. .00%) and mood (negative vs. neutral) and employed computational modeling to identify underlying mechanisms. Results showed that alcohol intoxication impaired IGT performance, with intoxicated participants selecting fewer cards from advantageous decks (estimate=−8.12, 95% CI=[−12.83, −3.23]). Evidence for an effect of negative mood was moderate but inconclusive (estimate=−4.82, 95% CI=[−9.66, 0.02]). Computational modeling revealed that both alcohol (estimate=.13, 95% CI=[.05, .21]) and negative mood (estimate=.12, 95% CI=[.04, .20]) increased reward learning rates without affecting punishment learning rates. No interaction effects were observed. These findings suggest that impaired decision-making during alcohol intoxication and negative mood states stems from heightened sensitivity to immediate rewards rather than diminished sensitivity to punishments but these effects do not appear to be additive, providing novel insights into the computational mechanisms underlying alcohol-related decision-making deficits.

## 1. Introduction

The Iowa Gambling Task (IGT) is a widely used paradigm for assessing decision-making under uncertainty, where participants must learn through experience to select from advantageous versus disadvantageous decks of cards (Bechara et al., 1994, 2000). Success on the IGT requires individuals to learn from patterns of rewards and punishments to identify which decks yield favorable long-term outcomes. Specifically, participants must distinguish between decks with different reward-punishment structures: two decks (A and B) offer high immediate rewards but larger penalties (varying in size and frequency), yielding net losses in the long run, while two decks (C and D) provide more modest immediate rewards but smaller penalties (also varying in size and frequency), yielding net gains in the long run. The ability to effectively weigh immediate rewards against potential negative consequences, as measured by the IGT, is fundamental to adaptive decision-making across numerous contexts (Dayan and Daw, 2008; Dora and Inzlicht, 2025; Joyner et al., 2019; Wichers et al., 2015).

Research using the IGT has consistently demonstrated that decision-making processes can be disrupted in various clinical populations. For instance, individuals with alcohol use disorder (AUD) tend to make more selections from disadvantageous decks compared to controls (Grant, 2000; Kovács et al., 2017; Logge et al., 2023). Similar decision-making impairments on the IGT have been documented in individuals with major depressive disorder (Han et al., 2012; Wang et al., 2024), eating disorders (Guillaume et al., 2015), and gambling disorders (Kovács et al., 2017). These findings highlight that both addiction-related conditions and affective disorders are associated with alterations in how individuals process and learn from reward and punishment information in the context of decision-making.

While these between-group comparisons have established important associations between clinical conditions and IGT performance, they are limited in their ability to elucidate the underlying mechanisms of decision-making impairments. Furthermore, they cannot address how decision-making processes are affected by transient states, such as acute alcohol intoxication or temporary mood fluctuations, which may be particularly relevant for understanding momentary decision-making vulnerabilities. Moving beyond trait-level comparisons to examine state-level effects offers an opportunity to directly test causal mechanisms underlying decision-making alterations.

Alcohol consumption is known to significantly alter cognitive processes that are essential for adaptive decision-making. Alcohol myopia theory (Steele and Josephs, 1990) provides a framework for understanding these effects, proposing that alcohol narrows attentional focus to the most salient cues in the environment while diminishing the processing of more peripheral or distal information. This attentional narrowing may have implications for how individuals process reward and punishment information during decision-making tasks. Laboratory studies have demonstrated that alcohol consumption leads to increased risk-taking and altered information processing (Dora et al., 2025; George et al., 2005; McMullin et al., 2022; Neilson et al., 2024; Steele and Josephs, 1990; Van Ravenzwaaij et al., 2012). However, the specific computational mechanisms through which alcohol affects the processing of rewards versus punishments remain poorly understood.

The IGT provides a structured context to examine these cognitive mechanisms in detail, as it requires integrating both immediate reward information and long-term punishment consequences, which are precisely the types of processes that might be differentially affected by alcohol according to myopia theory. Despite the established effects of alcohol on various domains of decision-making and information processing, surprisingly, no studies to date have directly examined how acute alcohol intoxication affects IGT performance and its underlying computational processes. Understanding these effects would provide valuable insights into the specific decision mechanisms disrupted during alcohol intoxication.

Similarly, emotional states are thought to influence decision-making (Blanchette and Richards, 2010; Shenhav, 2024), as stress and negative emotions have been found to influence how people evaluate risk (Clark et al., 2001), make judgments (Clore and Huntsinger, 2007), invest effort (Byrne et al., 2023), and make alcohol-related decisions (Bresin et al., 2018; Dora et al., 2023a, 2024). Laboratory studies examining the effects of negative mood and stress inductions on IGT performance have found that people tend to perform worse when experiencing negative emotional states or stress (Ben Hassen et al., 2023; Buelow and Suhr, 2013; Chrusciel et al., 2024; De Vries et al., 2008; Molins et al., 2025; Simonovic et al., 2017; Van Den Bos et al., 2009; Wemm and Wulfert, 2017), though occasionally null results have also been reported (Buelow et al., 2013; Clark et al., 2001; Shukla et al., 2019). Like alcohol, negative affect may change how individuals allocate attention to and process different aspects of decision-relevant information (Bishop and Gagne, 2018).

Further, investigating the potential interaction between alcohol intoxication and negative emotional states on decision-making processes is theoretically important for several reasons. First, both states may independently affect similar attentional and information processing mechanisms but potentially through different pathways. Second, their co-occurrence might be a unique decision-making context as people frequently cite negative emotions as a major motivation for alcohol use (Cooper et al., 1995; Hammarberg et al., 2017; Kuntsche et al., 2008), though it is unclear whether people regularly drink alcohol in response to negative affect (Dora et al., 2023b). From a theoretical perspective, alcohol’s attentional narrowing effects may interact with negative mood’s influence on information processing in ways that could either compound or counteract each other. For high-risk drinkers, understanding this interaction could provide insights into how these states might alter the ability to appropriately weigh immediate rewards against potential negative consequences, a process that may contribute to problematic drinking patterns.

Traditional IGT scoring, which simply counts deck selections, provides limited insight into the specific decision-making processes involved. For example, poor IGT performance could result from heightened sensitivity to rewards, diminished sensitivity to punishments, or both, distinctions that have significant theoretical implications but remain undetectable with conventional scoring methods. Computational modeling approaches, such as the Outcome-Representation Learning model (ORL; Haines et al., 2018; Sullivan-Toole et al., 2022), address this limitation by explicitly modeling different decision strategies and learning parameters. The ORL model explicitly accounts for four key cognitive strategies observed in IGT performance. Expected value maximization involves learning that decks C and D yield better long-term outcomes than decks A and B, despite A and B offering larger immediate rewards; the model captures this through separate expected value learning for each deck using distinct reward and punishment learning rates. Gain-loss frequency sensitivity captures the strong preference many participants show for decks B and D (which win on 90% of trials) over decks A and C (which win on only 50% of trials), even when this preference is disadvantageous; the model tracks this through a separate expected frequency component that monitors how often each deck provides wins versus losses. Choice perseveration reflects whether participants tend to stick with recently chosen decks or switch away from them, regardless of the outcomes received; the model includes a perseveration component that weights recently chosen options more heavily in future decisions. Reversal learning involves quickly changing deck preferences after experiencing unexpected large losses, such as switching away from deck B after encountering its large punishment; the model accomplishes this by updating gain-loss frequency expectations for unchosen decks based on outcomes observed from chosen decks.

Two particularly important parameters in the ORL model are the reward learning rate and punishment learning rate. These parameters reflect how much participants update their expectations about a deck after receiving positive versus negative outcomes. For example, a participant with a high reward learning rate would quickly develop strong preferences for decks that provide several wins in a row, rapidly updating their expectations upward after each positive outcome. Conversely, a participant with a high punishment learning rate would quickly learn to avoid decks after experiencing losses, substantially updating their expectations downward after negative outcomes. A participant who shows poor IGT performance due to chasing immediate rewards might have a high reward learning rate but low punishment learning rate; they quickly learn to like decks that give frequent wins but are slow to learn from the large losses that eventually follow. Conversely, a participant with a high punishment learning rate but low reward learning rate would quickly learn to avoid decks after experiencing losses but be slow to develop preferences for consistently rewarding options, potentially leading to overly cautious decision-making that misses good opportunities. In their paper validating the ORL, (Haines et al., 2018) found that individuals with a history of heroin use showed lower punishment learning rates than controls, suggesting reduced sensitivity to negative outcomes, while cannabis users demonstrated increased reward learning rates. These parameter-level insights go beyond traditional scoring approaches to reveal specific computational mechanisms underlying impaired decision-making.

### 1.1. Current study

Building on this literature, the current study aims to investigate the individual and combined effects of alcohol administration and a negative mood induction on IGT performance in a sample of adults who drink heavily. By experimentally manipulating both alcohol intoxication and negative mood in a controlled setting and applying computational modeling to the resulting data, we can identify specific decision-making processes affected by these states. This approach allows us to test competing theoretical accounts: Does alcohol primarily enhance reward sensitivity, diminish punishment sensitivity, or affect both? Does negative mood operate through similar or different computational mechanisms? And critically, do these states interact in ways that might be particularly relevant for understanding decision-making in high-risk drinkers?

We hypothesized that both alcohol intoxication and negative mood will result in fewer choices from the winning decks, with potential interactive effects when combined. We further predicted that these manipulations would affect reward and punishment learning rates as captured by the ORL model, with alcohol and negative mood increasing reward sensitivity while decreasing punishment sensitivity, providing insight into the computational mechanisms underlying decision-making under uncertainty.

## 2. Methods

### 2.1. Transparency

We preregistered design, hypotheses, sample size, modeling, and statistical analyses prior to data collection. All subsequently described decisions were preregistered in detail unless noted otherwise. Our preregistration, experimental materials, anonymized data, and analysis scripts are available on the Open Science Framework (osf.io/ky3aj/).

### 2.2. Participants and design

We determined our sample size through an a priori power analysis, which indicated that data from 160 participants (40 per condition) would provide 87.86% power to detect a medium-sized interaction effect (*η*^2^=.06) for our 2×2 between-subjects design. An overview of participant demographics can be found in Table 1. The median Alcohol Use Disorder Identification Test score of the sample was 8 (*mean=*9.38, *SD=*4.58, *range=*2–22), indicating that this can be considered a high-risk drinking sample. We recruited adults from the Seattle community via advertisement in public places and online ads. Participants were compensated at a rate of $25/hour. Participants had to be 21–50 years old and report regular alcohol use (≥1 drinking episode/week and ≥1 binge episode [i.e., 4/5 drinks for women/men in one occasion]/month). We excluded individuals who were currently pregnant or trying to become pregnant, had current/prior alcohol treatment, had past or current diagnosis of an anxiety disorder, or had medical conditions or took medications that contraindicate alcohol use. The study procedures were reviewed and approved by the University of Washington Institutional Review Board.

### 2.3. Procedure

#### 2.3.1. Laboratory session

Upon responding to advertisements, participants completed an online screening survey to check eligibility criteria, which was confirmed via phone. Eligible participants were randomly assigned to one of four conditions using block randomization: alcohol and negative mood, no alcohol and negative mood, alcohol and neutral mood, or no alcohol and neutral mood. All sessions started between 2:00pm and 5:00pm. Participants first provided informed consent, were breathalyzed to ensure their Breath Alcohol Concentration (BrAC) was .00%, and confirmed that they did not consume any calories for three hours prior to the laboratory session. Participants assigned female sex at birth completed a pregnancy test if necessary, as determined by screening questions administered by a female research assistant to ensure both medical necessity and respect for gender identity, and all participants were weighed. The total laboratory lasted approximately 45–60 minutes consisting of beverage administration (9 minutes consumption + 8–25 minutes for BrAC monitoring), mood induction (~5 minutes), and the Iowa Gambling Task (5–10 minutes). Participants completed the laboratory session following this sequence:

1. Beverage administration: All participants were seated in a room simulating a bar environment,including a wet bar, bar stools, liquor bottles, and bar paraphernalia. Participants randomly assigned to the alcohol condition were informed that they would receive alcohol and administered 100-proof Vodka mixed with orange juice (1:3 ratio) to induce a breath alcohol concentration (BrAC) of .06% (based on Widmark’s formula). The protocol involved consuming three equal portions of the beverage over 9 minutes total, with 3 minutes allocated per cup and pacing instructions provided by the research assistant to prevent rapid consumption. BrAC. Participants in the no alcohol condition were informed that they would not receive alcohol and administered water mixed with orange juice (1:3 ratio). Following beverage consumption, all participants completed a standardized mouth-rinsing protocol (5 mouth rinses followed by 1 gargle) to ensure accurate breathalyzer readings by removing residual alcohol from the mouth. Participants were then breathalyzed every 5 minutes until reaching peak BrAC. Each participant in the no alcohol condition was yoked to a participant in the alcohol condition, spending the same amount of time in the bar laboratory and completing the same number of breath tests. All participants reported their subjective intoxication (1=‘not at all intoxicated’, 10=‘extremely intoxicated’) immediately prior to leaving the bar laboratory.
2. Mood induction: Participants were moved to a different room and asked to look at 20 photographsfor 10 seconds each from the International Affective Picture Set (IAPS; Lang et al., 2020). They were instructed to try to become aware of and feel the emotions they experience while looking at the pictures as vividly as possible. Participants in the negative mood condition viewed photographs with a mood valence rating of <2.5 and arousal rating of <6. Those in the neutral mood condition viewed photographs with a mood valence rating between 4.5 and 5.5 and an arousal rating of <6. Participants rated their mood (1=‘extremely unhappy’, 10=‘extremely happy’) and stress level (1=‘not at all stressed’, 10=‘extremely stressed’) before and after the mood induction procedure.
3. Iowa Gambling Task: Participants completed the original (computerized) version of the IGT. For 100 trials, they freely drew from one of four decks, trying to maximize their earnings. Cards from each deck yielded a monetary gain and sometimes a monetary loss. Each deck had a different and fixed payout distribution, with two decks (A+B) being disadvantageous and two (C+D) being advantageous in the long run.

### 2.4. Analysis plan

#### 2.4.1. Computational modeling

To compute reward and punishment learning rates, we fitted the ORL model (Haines et al., 2018) to the IGT data via the hBayesDM package (Ahn et al., 2017). The ORL model assumes separate learning rates for positive and negative outcomes, with reward learning rate and punishment learning rate estimated across trial-level computations for both expected value and expected frequency of outcomes, as follows:

Expected value is computed as follows:

For positive outcomes (when x(t) ≥ 0):

$$EV_{j(t+1)}=EV_{j(t)}+A_{\text{rew}}*\left(x(t)-EV_{j(t)}\right)$$

For negative outcomes:

$$EV_{j(t+1)}=EV_{j(t)}+A_{\text{pun}}*\left(x(t)-EV_{j(t)}\right)$$

where EV_j(t)_ is the expected value of deck j on trial t, and x(t) is the experienced outcome. A_rew_ and A_pun_ (both bounded between 0 and 1) are learning rates for rewards and punishments, respectively, that are estimated across the trial-level computations for EV and EF (see below).

Expected win frequency for the chosen deck is computed as follows:

For positive outcomes (when x(t) ≥ 0):

$$EF_{j(t+1)}=EF_{j(t)}+A_{\text{rew}}*\left(\text{sgn}(x(t))-EF_{j(t)}\right)$$

For negative outcomes:

$$EF_{j(t+1)}=EF_{j(t)}+A_{\text{pun}}*\left(\text{sgn}(x(t))-EF_{j(t)}\right)$$

where EF_j(t)_ is the expected win frequency of the chosen deck j on trial t, and sgn(x(t)) returns 1, 0, or −1 for positive, zero, or negative outcome values on trial t, respectively. Expected frequency is also updated across all unchosen decks using a reversal learning computation (Haines et al., 2018).

The model also includes a perseverance component PS that captures the tendency to repeat or switch away from the previous deck choice, regardless of outcome:

where the chosen deck is D_(t)_ = j:

$$PS_{j(t+1)}=\frac{1}{1+K}$$

And for the unchosen decks as

$$PS_{j(t+1)}=\frac{PS_{j}}{1+K}$$

where K = 3^K’^ – 1, and K’ is a parameter controlling memory decay for the history of choices made.

The combined value of each deck is determined by

$$V_{j(t+1)}=EV_{j(t+1)}+EF_{j(t+1)}*\beta F+PS_{j(t+1)}*\beta P$$

where V_j(t)_ is the value of deck j on trial t, *β*F is the win frequency sensitivity parameter (higher values indicate greater preference for decks with higher win frequency), and *β*P is the perseveration tendency parameter (higher values indicate greater choice consistency). These values are then entered into a softmax function to generate choice probabilities:

$$P\left[\left(D_{\left(t+1\right)}\right)=j\right]=\frac{e^{V_{j(t+1)}}}{\sum_{{k=1}^{e^{V_{k}(t+1)}}}^{4}}$$

where D(t) represents the chosen deck on trial t.

In summary, the ORL model has five free parameters: the reward learning rate A_rew_ and punishment learning rate A_pun_ reflect the rate at which an individual updates EV and EF for a given deck following gains and losses, respectively; the win frequency sensitivity *βF* reflects the preference for decks with a higher win frequency over equivalent decks that win less often; the perseveration tendency *βP* reflects a preference to stick with a previous choice; and the memory decay K reflects the extent to which an individual forgets their own history of choices.

#### 2.4.2. Statistical models

For all hypotheses, we fitted Bayesian models using the brms package (Bürkner, 2017) with weakly informative priors. We confirmed that the models converged via Rhat statistics, effective sample sizes, and by inspecting trace plots. We made sure that models fitted the data via posterior predictive checks. We fitted three preregistered models:

1. A linear model including main effects for alcohol intoxication and mood induction and their-interaction predicting the choice of winning decks in the IGT (using the final 60 trials; Gansler et al., 2011).
2. A linear model including main effects for alcohol intoxication and mood induction and their-interaction predicting the reward learning rate.
3. A linear model including main effects for alcohol intoxication and mood induction and their-interaction predicting the punishment learning rate.

We preregistered two sensitivity analyses. First, we repeated our analyses excluding participants who did not respond to the mood induction as expected (less than 3-point decrease in the negative mood condition, at most 2-point change in either direction in the neutral mood condition). Second, we repeated our analyses with default brms priors.

#### 2.4.3. Inference criteria

We report the full posterior distribution for each parameter of interest. For H1, we defined a region of practical equivalence (ROPE) (Kruschke, 2018) as a difference of ±3 good draws. For H2 and H3, as we had no a priori expectation about meaningful effect sizes for the ORL parameters, we interpret any 95% CI that excludes 0 as evidence for an effect of our experimental manipulations.

## 3. Results

### 3.1. Descriptive statistics

**Figure 1.**
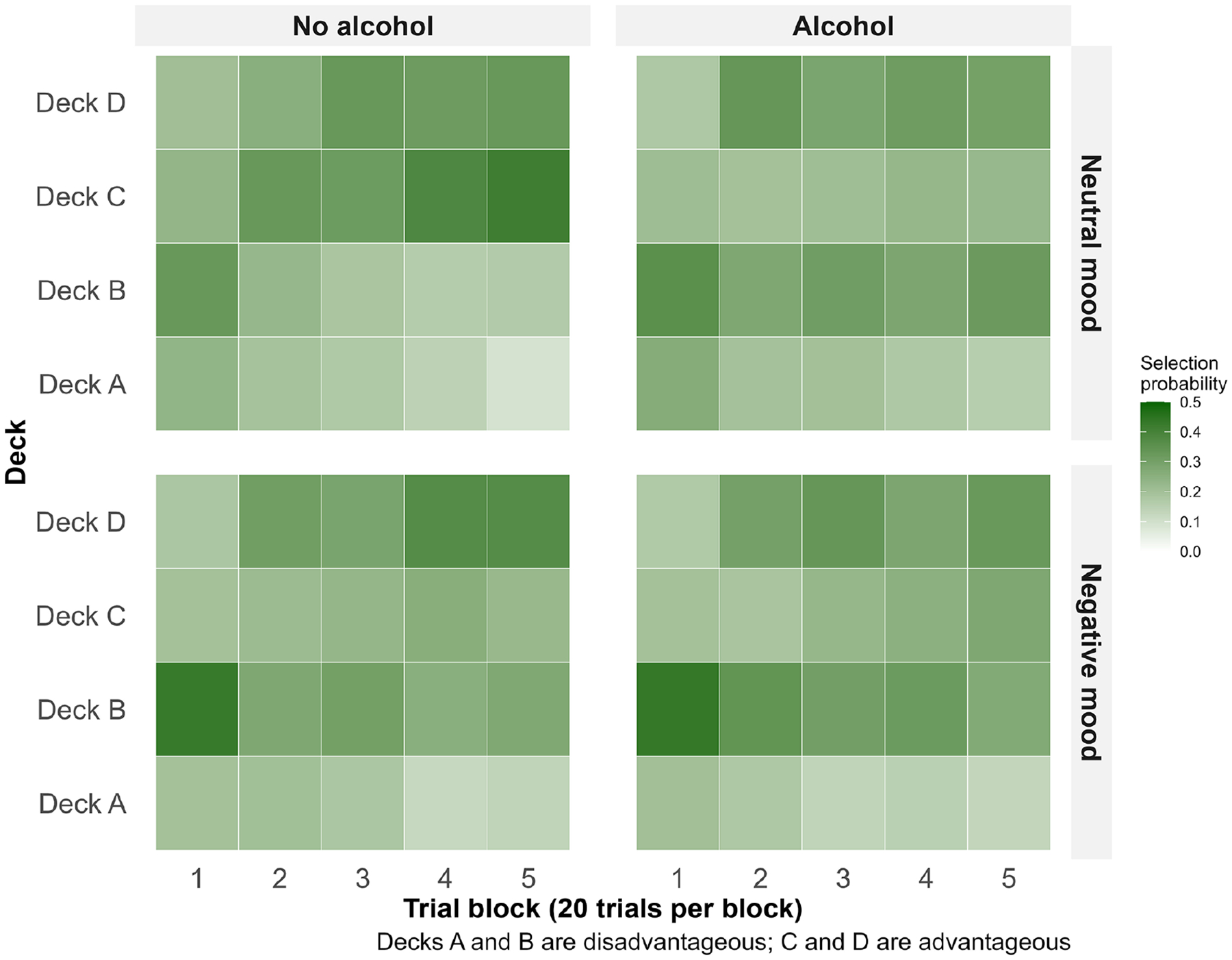
Heatmap of deck preference evolution by experimental condition.

On average, participants drew 35.33 cards from the two winning decks in the final 60 trials (*SD*=13.11). Across all 100 trials, participants chose least often from Deck A (17.2%), followed by Deck C (24.9%), Deck D (28.8%), and Deck B (29.0%). Figure 1 shows the evolution of deck selections over the course of the task split by experimental condition. As expected, reward learning rate was negatively correlated with performance (*r*=−0.32), and punishment learning rate was positively correlated with performance (*r*=0.50).

### 3.2. Manipulation check

#### 3.2.1. Alcohol intoxication

**Figure 2.**
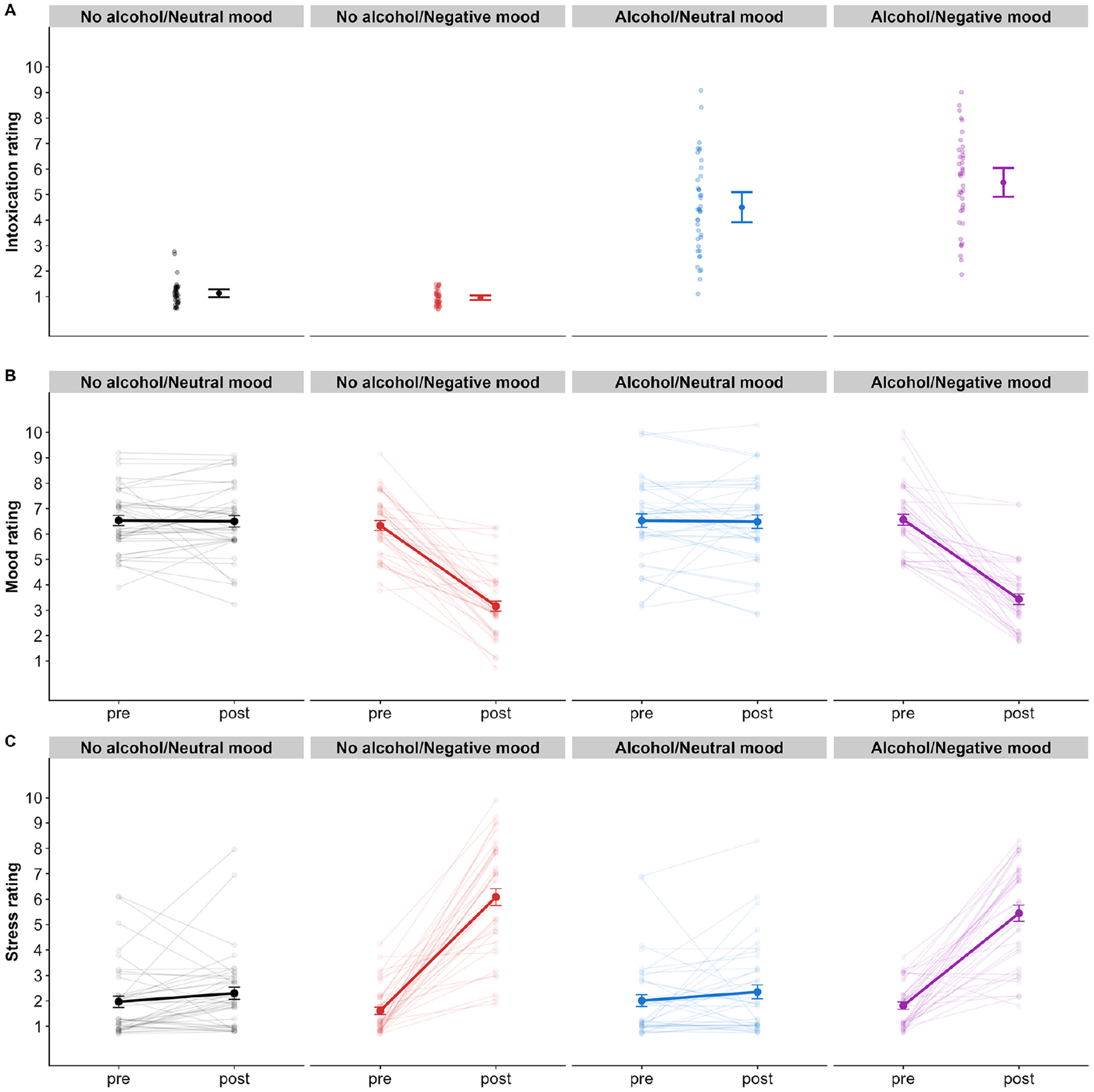
Manipulation checks. (a) Self-reported intoxication. (b) change in self-reported mood. (c) change in self-reported stress. Error bars represent standard errors.

Target BrAC (.06% in alcohol conditions, .00% in no alcohol conditions) was confirmed via breathalyzer just prior to the mood induction. Participants in the alcohol conditions achieved actual BrACs very close to the target, with those in the alcohol/negative mood condition reaching a mean BrAC of .060% (*SD* = .006, *range* = .052–.072) and those in the alcohol/neutral mood condition reaching a mean BrAC of .058% (*SD* = .006, *range* = .049–.072). Participants in the alcohol conditions reported higher subjective intoxication (*M*=4.96, *SD*=1.85, *range*=1–9; *Cohen’s d*=2.93) than participants in the no alcohol conditions (*M*=1.06, *SD*=0.33, *range*=1–3; Figure 2a).

#### 3.2.2. Mood

Following the mood induction, participants in the negative mood conditions reported a decrease in mood (*M*=−3.19, *SD*=1.76, *range*=−8–1; *Cohen’s d*=2.04) and an increase in stress (*M*=4.05, *SD*=2.26, *range*=−1–8; *Cohen’s d*=1.88), while mood and stress levels remained stable in the neutral mood conditions (*M*_ΔMood_=−0.01, *SD*_ΔMood_=1.32, *range*_ΔMood_=−4–5, Figure 2b; *M*_ΔStress_=0.35, *SD*_ΔStress_=1.62, *range*_ΔStress_=−6–5, Figure 2c).

### 3.3. Preregistered analyses

#### 3.3.1. Behavioral data

**Figure 3.**
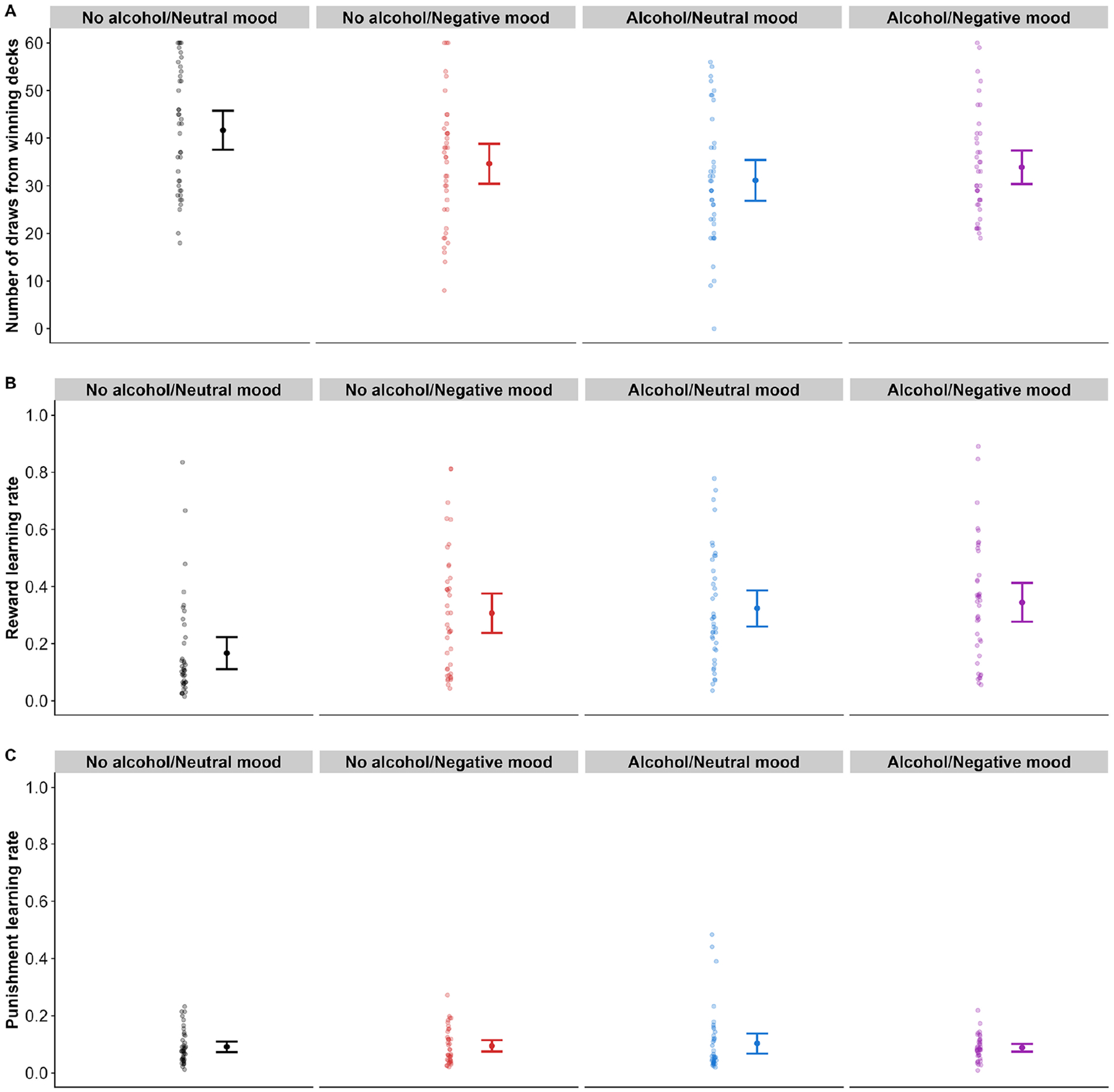
Behavioral data and computational parameters. (a) Number of draws from winning decks (C+D) in the final 60 trials. (b) Reward learning rate derived from ORL model. (c) Punishment learning rate derived from ORL model. Error bars represent standard errors.

To test whether alcohol intoxication and negative mood affect IGT performance, we predicted the number of draws from winning decks in the final 60 trials. Our Bayesian model estimated that intoxicated participants on average draw 8.12 fewer cards from winning decks compared to sober participants (95% CI=[−12.83, −3.23]). As the interval falls entirely outside of our preregistered region of practical equivalence, we conclude that there is clear evidence in the data for a detrimental effect of alcohol intoxication on performance. The posterior distribution for the main effect of negative mood had a mean of −4.82 (95% CI=[−9.66, 0.02]). This can be considered moderate but inconclusive evidence for an effect of negative mood on performance, as 23.1% of the posterior draws fell within the ROPE. There was no evidence for an interaction between alcohol intoxication and negative mood (*estimate*=6.09, 95% CI=[−0.41, 12.64]; Figure 3a). The results were robust to the exclusion of participants who did not respond to the mood induction, the use of default brms priors, and were not affected when controlling for sex differences across conditions^1^.

#### 3.3.2. Computational modeling parameters

To validate that the ORL model provided a meaningful account of participants’ choice behavior, we compared model fit to a baseline random choice model using Leave-One-Out Information Criterion (LOOIC). The ORL model demonstrated substantially superior fit compared to random choice (ΔLOOIC = 228,693.6), with better individual-level fit for 159 out of 160 participants (99.4%). This indicates that the ORL model captures systematic patterns in decision-making behavior rather than noise, providing a valid foundation for interpreting the computational parameters. We predicted the reward and punishment learning rate derived from the ORL model. The parameters by experimental condition are summarized in Table 2. Our Bayesian model estimated that both alcohol (estimate=.13, 95% CI=[.05, .21]) and negative mood (estimate=.12, 95% CI=[0.04, .20]) predicted an increased reward learning rate, but there was no evidence for an interaction between the two (estimate=−.08, 95% CI=[−.19, .03]; Figure 3b). Neither alcohol (estimate=.01, 95% CI =[−.02, .04]) nor negative mood (estimate=.00, 95% CI=[−.03, .04]) or their interaction (estimate=−.02, 95% CI=[−.06, .03]; Figure 3c) predicted the punishment learning rate. These results were robust to our three sensitivity analyses.

## 4. Discussion

The current study examined how alcohol intoxication and negative mood, both independently and in combination, affect decision-making processes during the Iowa Gambling Task in a high-risk sample of regularly drinking adults. Our findings provide novel insights into the computational mechanisms underlying decision-making while intoxicated and in a negative mood.

Our results demonstrated a clear detrimental effect of moderate alcohol intoxication on IGT performance, with intoxicated participants making fewer selections from winning decks compared to sober participants. This finding can be interpreted through the lens of alcohol myopia theory (Steele and Josephs, 1990), which posits that alcohol narrows attention which results in the amplification of immediate, salient cues while diminishing the processing of more peripheral cues. Based on our computational modeling results, it seems that in the context of the IGT alcohol increases the sensitivity to immediate rewards without decreasing the sensitivity to large infrequent punishments. The fact that alcohol appears to affect the reward learning rate but not punishment learning rate may help explain why intoxicated individuals may appear ‘myopic’ during decision-making; they are not necessarily less sensitive to negative consequences, but rather disproportionately influenced by immediate rewards. These results extend previous behavioral observations by providing a computational mechanism through which decision-making is impaired when intoxicated.

Our behavioral findings align with earlier studies that showed that people tend to make riskier choices following alcohol administration (George et al., 2005, 2009), though those studies could not distinguish whether risk-taking was due to changes in the processing of rewards, or punishment, or both. Our computational approach provides this mechanistic insight. They also align with neurobiological evidence that consuming alcohol promotes the release of dopamine in brain regions involved in the processing of rewards (Boileau et al., 2003; Gilman et al., 2008). The specific effect on reward learning rate without corresponding changes in punishment learning reveals a more nuanced picture of alcohol’s impact on decision processes than previously understood.

Our study suggests moderate but inconclusive evidence for an effect of negative mood on IGT performance. While participants in the negative mood conditions tended to make fewer selections from advantageous decks, the wide credible interval reflects substantial uncertainty in this estimate. This inconclusiveness mirrors the mixed literature on negative mood and IGT performance, where some studies report detrimental effects (Ben Hassen et al., 2023; Buelow and Suhr, 2013; Chrusciel et al., 2024; De Vries et al., 2008; Molins et al., 2025; Simonovic et al., 2017; Van Den Bos et al., 2009; Wemm and Wulfert, 2017) while others reported null results (Buelow et al., 2013; Clark et al., 2001; Shukla et al., 2019). Despite this behavioral ambiguity, our computational modeling provided clearer evidence, showing that negative mood, like alcohol intoxication, increased reward learning rates without affecting punishment learning rates.

Our design does not allow us to infer conclusively why the results for alcohol intoxication and negative mood are so similar. One potential explanation is that the parallel computational effects of alcohol and negative mood on reward learning, despite their distinct physiological mechanisms, might suggest a potentially shared information processing pathway. Both states may influence attention allocation in ways that heighten the salience of immediate rewards relative to potential future punishments. (Bishop and Gagne, 2018)’s computational framework helps contextualize these findings, as they propose that affective states influence which outcomes are mentally sampled during decision processes. Our results suggest that both alcohol and negative mood may alter this sampling process in similar ways, specifically by increasing the weight given to reward information during expectation updating, without necessarily diminishing punishment sensitivity.

Our findings show agreement with Laycock et al. (2024), who also used the ORL model and similarly found that negative states resulted in decreased behavioral performance and increased sensitivity to rewards but not punishment through computational modeling. Our results also align conceptually with Lane et al. (2006), who used the Expectancy-Valence model. They found no difference in their unvalenced learning rate parameter, which collapses across both positive and negative outcomes, but did find that alcohol increased sensitivity to gains while decreasing sensitivity to losses through their motivation parameter. This aligns with our finding of increased reward learning rates when intoxicated, suggesting that both computational approaches may be capturing similar underlying mechanisms, alcohol’s enhancement of positive outcome processing, but through different parameterizations. The alignment occurs with their motivation parameter rather than learning parameter because their unvalenced learning rate would mask the reward-specific learning effects that our separate reward and punishment learning parameters can detect. However, our findings contrast with those of Gullo and Stieger (2011), who also used the Expectancy-Valence model but reported that anticipatory stress improved IGT performance in regular alcohol drinkers. Their computational modeling showed that stress increased attention to losses, whereas we found that negative mood increased reward learning rates without affecting punishment learning rates. The discrepancy between our behavioral findings and those of Gullo and Stieger is puzzling, given that our study and theirs used similar inclusion criteria and our manipulation resulted in increased stress in addition to decreased mood.

Our data were not in line with our prediction that alcohol and negative mood would have synergistic effects on decision-making. We found no evidence for an interaction between these manipulations on either behavioral IGT performance or computational parameters. This finding is particularly interesting given that both manipulations independently produced similar computational effects. The absence of an interaction suggests that alcohol and negative mood may affect reward sensitivity in similar but non-additive ways, potentially reaching ceiling effects on the same underlying cognitive mechanisms.

While our study provides novel insights into how alcohol intoxication and negative mood affect decision-making, several limitations should be acknowledged. First, our study used a single moderate dose of alcohol (target BrAC of .06%), which may not capture the full range of alcohol’s effects on decision-making. Future studies could examine dose-dependent relationships between alcohol and computational parameters (Sher and Walitzer, 1986), including testing the interaction with negative mood at higher intoxication levels. Second, our sample consisted of adults who drink regularly but have no AUD diagnosis. It would be interesting to compare such a group to either individuals who drink less regularly and/or individuals with AUD, as previous work has shown baseline differences in IGT performance in this population (Kovács et al., 2017). Further, it would be valuable to explore whether our experimental manipulations affect IGT performance and learning rates in different ways across these groups.

Third, our mood induction procedure has inherent limitations. The IAPS-based mood induction used low-to-moderate arousal negatively valenced images, and such mood effects might be short-lived. However, our experimental design was optimally configured temporally; participants completed the IGT immediately following the mood induction (within 5 minutes), and BrAC measurements confirmed participants remained at peak intoxication both before and after the IGT. Nevertheless, the transient nature of low-arousal mood manipulations, combined with our moderate alcohol dose, may explain why we observed no additive effects of alcohol and negative mood on reward learning rates. Future studies should consider more sustained mood manipulations or higher-arousal negative states that might produce longer-lasting effects.

Fourth, it is unclear whether the results are contingent on the timing on the BAC curve. Past studies have reported differential effects of alcohol intoxication on related constructs such as risk taking, executive control, and memory on the ascending versus descending limb (Davis et al., 2009; Jones, 1973; Pihl et al., 2003). Therefore, it is possible that alcohol can affect decision-making more or less than is reported here depending on whether a person is on the ascending or descending limb. Our findings at peak BAC represent the maximum pharmacological effect, but future research examining decision-making across different phases of the alcohol curve could provide additional insights into the temporal dynamics of alcohol’s effects on reward and punishment processing.

Fifth, and importantly, this laboratory-based study has limited ecological validity. While the IGT is widely used to assess decision-making, there is limited evidence that performance on this task reflects real-world decision-making processes. The task involves artificial rewards and punishments in a controlled setting, which may not generalize to complex real-world decisions that involve stronger emotions, contextual influences, and more meaningful consequences. Future research should bridge this gap by combining laboratory tasks with more naturalistic approaches, such as ecological momentary assessment of decision-making in daily life, to better understand how alcohol and negative mood influence decisions in contexts with higher ecological validity. An alternative would be to assess connections between IGT performance and self-reported alcohol-related attitudes or real-world drinking behaviors, which would help us to understand how laboratory-based decision-making might translate to actual alcohol use patterns or problems.

In conclusion, our study suggests that decision-making involving a tradeoff between rewards and punishment is most likely impaired when one is intoxicated at a BrAC of .06%, possibly impaired when in a negative mood, but unlikely to be further impaired by both intoxication and negative mood. By demonstrating that both alcohol intoxication and negative mood specifically increase reward sensitivity without affecting punishment sensitivity, our study has an important practical implication: decision-making vulnerabilities during drinking may stem from amplified attraction to immediate rewards rather than inability to learn from negative consequences. A promising avenue for the future is to examine whether these computational signatures can predict real-world risky alcohol-related decision-making.

## Figures and Tables

**Table 1.** Participant characteristics split by experimental condition.

	No alcohol/neutral mood	No alcohol/negative mood	Alcohol/neutral mood	Alcohol/negative mood
Age	M=30.08, SD=7.97	M=30.18, SD=7.19	M=32.30, SD=7.33	M=31.43, SD=8.02
Sex	27.5% female,	60.0% female,	25.0% female,	62.5% female,
	72.5% male	40.0% male	75.0% male	37.5% male
Gender	21.1% cisgender woman,	57.5% cisgender woman,	25.0% cisgender woman,	55.0% cisgender woman,
	76.3% cisgender man,	35.0% cisgender man	70.0% cisgender man	37.5% cisgender man,
	2.6% gender-expansive	7.5% gender-expansive	5.0% gender-expansive	7.5% gender-expansive
Race/ethnicity	12.5% Asian,	17.5% Asian,	7.5% Asian,	20.0% Asian,
	25.0% Black/African American,	5.0% Black/African American,	20.0% Black/African American,	2.5% Black/African American,
	45.0% white	52.5% white,	35.0% white,	50.0% white,
	5.0% multiracial;	15.0% multiracial;	12.5% multiracial;	15.0% multiracial;
	12.5% Hispanic/Latino	10.0% Hispanic/Latino	20.0% Hispanic/Latino	12.5% Hispanic/Latino
Sexuality	22.5% LGBQ,	44.7% LGBQ,	15.4% LGBQ,	29.7% LGBQ,
	77.5% straight	55.3% straight	84.6% straight	70.3% straight
Student status	10.0% students	20.5% students	18.4% students	22.5% students
Employment	32.5% unemployed,	15.0% unemployed,	15.0% unemployed,	20.0% unemployed,
	30.0% part-time,	35.0% part-time	37.5% part-time,	27.5% part-time,
	37.5% full-time	50.0% full-time	47.5% full-time	52.5% full-time
AUDIT	M=9.59, SD=4.84	M=10.10, SD=4.63	M=9.81,SD=4.28	M=8.08, SD=4.46

*Note*: Gender-expansive includes transgender men, transgender women, and non-binary participants. LGBQ includes gay, lesbian, bisexual, pansexual, asexual, queer, and questioning/not sure participants.

**Table 2.** ORL model parameters by experimental condition.

Condition	Reward learning rate *M (SD)*	Punishment learning rate *M (SD)*
No alcohol/neutral mood	0.167 (0.175)	0.091 (0.058)
No alcohol/negative mood	0.307 (0.215)	0.095 (0.061)
Alcohol/neutral mood	0.324 (0.198)	0.103 (0.110)
Alcohol/negative mood	0.345 (0.212)	0.088 (0.042)

## Data Availability

Processed de-identified participant data and analysis scripts are available at osf.io/ky3aj/.

## Footnotes

1 These results are summarized in a RMarkdown and html document on the OSF project page.
